# The clinical effect of nicorandil on perioperative myocardial protection in patients undergoing elective PCI: A Systematic Review and Meta-Analysis

**DOI:** 10.1038/srep45117

**Published:** 2017-03-21

**Authors:** Ziliang Ye, Qiang Su, Lang Li

**Affiliations:** 1 Department of cardiology, The First Affiliated Hospital of Guangxi Medical University, Guangxi Cardiovascular institue, Nanning, Guangxi, 530021, China

## Abstract

Many scholars have studied the effect of nicorandil on perioperative myocardial protection in patients undergoing elective percutaneous coronary intervention (PCI), but results are inconsistent. Therefore, we performed this meta-analysis. Finally, 16 articles, including 1616 patients, were included into this meta-analysis. Meta-analysis results showed that: (1) Nicorandil can reduce the level of CK-MB after PCI, including at 6 hours, 12 hours, 18 hours and 24 hours. (2) Nicorandil can reduce the level of TnT after PCI, including at 6 hours, 12 hours, 18 hours and 24 hours. (3) Nicorandil can reduce the incidence of adverse reactions after PCI. (4) Nicorandil cannot reduce the level of MVP after PCI, including at 12 hours and 24 hours. (5) Subgroup analysis showed that nicorandil can reduce CK-MB and TnT level at 24 hours after PCI for Chinese’s population (P < 0.05), but can not reduce CK-MB and TnT level at 24 hours after PCI for non Chinese’s population (P > 0.05). Our meta-analysis indicate that nicorandil can reduce myocardial injury and reduce the incidence of adverse reaction caused by PCI for Chinese’s population, but is not obvious for non Chinese’s population. However, this conclusion still needs to be confirmed in the future.

In recent years, percutaneous coronary intervention (PCI)^1^,^2^,^3^ has become the principal means of revascularization in patients with coronary heart disease(CHD). According to new research, PCI can obviously improve the myocardial ischemia symptoms and reduce the incidence of cardiovascular events in patients by the revascularization of the ischemic area^4^,^5^. However, some complications will inevitably happen during PCI^6^. Perioperative myocardial injury (PMI) is one of the common complications to the PCI process, and the incidence rate is approximately 5~30%^7^,^8^. Many research results have indicated that the mechanism of myocardial injury after PCI include vascular endothelial injury, distal microvascular thrombosis, surgical occlusion of the blood vessels, coronary artery spasm, plaque displacement leading to side branch occlusion and reperfusion injury^9^,^10^. Once occurred PMI, the incidence of long-term adverse cardiovascular events will be significantly increased, and result in poor prognosis. Therefore, early identification, prevention and treatment of PMI have an important clinical significance.

At present, a number of studies^11^,^12^,^13^ about how to prevent perioperative myocardial injury after PCI had been carried out, and the majority of researches are focused on how to prevent myocardial injury after PCI through drug therapy. So far, the commonly used drugs include aspirin, clopidogrel, heparin, statins, beta blockers and calcium antagonists and so on, and statins is commonly used in clinical. Studies have indicated that long-term use of statins can improve the initial clinical symptoms of ACS patients and reduce the incidence of ST segment elevation myocardial infarction^14^,^15^. Different retrospective studies and meta-analysis have shown that for patients undergoing elective PCI, statin therapy can effectively reduce the incidence of postoperative PMI^16^,^17^,^18^. A large number of studies have shown that, in addition to stable plaque, statins can improve endothelial function and anti-inflammatory and improve the vascular wall inflammation induced by PCI. Meanwhile, it can effectively reduce the level of c-reactive protein, which can increase the survival rate of the patients after operation, and reduce the inflammatory reaction after PCI^19^,^20^.

However, statins also have some adverse drug reaction, such as liver toxicity and muscle toxicity^21^,^22^. The mechanism of liver toxicity induced by statin is not entirely clear, it may be related to the inhibition of HMG-CoA reductase and HMG-CoA pathway, leading to a reduction of the HMG-CoA reductase and metallic acid. *In vitro*, studies have revealed that statins can lead to apoptosis of liver cells, and fenofibrate can aggravate apoptosis. The typical clinical manifestations of muscle toxicity induced by statin are fatigue, muscle pain, muscle weakness, convulsions and tendon pain. From 1987 to 2001 years, the United States FDA recorded a total of 42 death cases of rhabdomyolysis induced by statins. Due to the incidence of adverse drug reaction induced by statins is increasing, many scholars are exploring new drugs to prevent the occurrence of perioperative myocardial injury after PCI.

Nicorandil is an anti-anginal agent with a dual mechanism of action. It is the only potassium channel opener which has the effect of antiangina pectoris, playing an effective role in the expansion of artery, vein and coronary artery^23^,^24^,^25^. In addition, there was no serious effect on heart rate, myocardial contractility and conduction system. However, the conclusion, whether nicorandil has a myocardial protective effect is inconsistent. Hwang J^26^ and Kim J^27^ have found that nicorandil had no significant effect on PMI and cardiac enzymes after PCI in patients with stable or unstable angina, but Kim S^28^, Murakami M^29^ and Shehata M^30^ have carried out studies about the clinical effect of nicorandi, and their conclusion is that nicorandil can significantly reduce myocardial enzymes in patients after PCI, which was different from Hwang J and Kim J. Therefore, the purpose of our meta-analysis is to evaluate the myocardial protective effect of nicorandil in the perioperative period of patients after PCI, and to provide a reference for clinical applications.

## Results

### Study Selection and Characteristics

**Figure 1:**
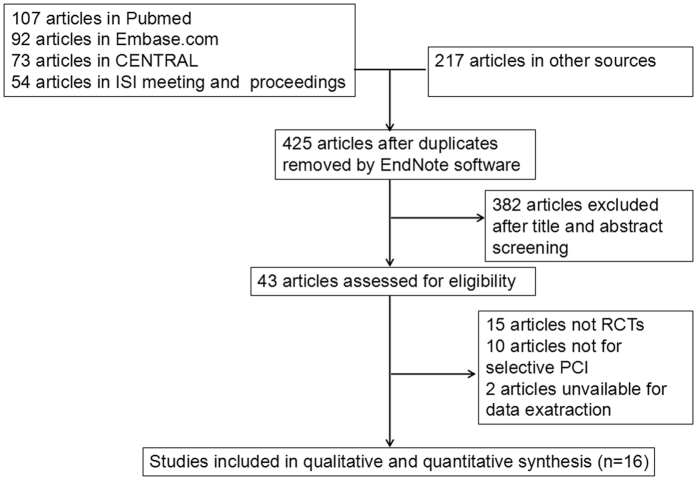
Flowchart of the selection strategy and inclusion/exclusion criteria in the current meta-analysis.

Finally, 543 articles were retrieval by searching the electronic databases and references of relevant articles. After excluding duplicate articles, 425 articles were left. By screening titles and abstracts of remaining articles, 382 apparently irrelevant articles were excluded. Then, the full texts of 43 articles were downloaded to evaluate in detail. Eventually, data from 16 articles^26^,^27^,^28^,^29^,^30^,^31^,^32^,^33^,^34^,^35^,^36^,^37^,^38^,^39^,^40^,^41^ include 1616 patients were listed into this meta-analysis. Among these patients, 804 patients in the nicorandil group and 812 patients in the control group. The flow diagram of study selection is shown in Fig. 1. The basic information of each included literature is shown in Table 1.

### Literature quality evaluation

Of the 16 articles, four articles^31^,^32^,^33^,^39^ used a random number method, three articles^28^,^30^,^41^ used a block randomization, eight articles^26^,^27^,^29^,^34^,^35^,^36^,^37^,^38^ refer to the random method, but did not give a specific description, and the random method of the rest article^40^ is not clear. The hidden distribution of the sixteen articles^26^,^27^,^28^,^29^,^30^,^31^,^32^,^33^,^34^,^35^,^36^,^37^,^38^,^39^,^40^,^41^ is low; Fifteen articles^26^,^27^,^28^,^29^,^30^,^31^,^32^,^33^,^34^,^35^,^36^,^37^,^38^,^39^,^41^ used a random single blind method, but the blind method of one article^40^ is not clear. The incomplete outcome data, selective reporting of results and other biases of the sixteen articles^26^,^27^,^28^,^29^,^30^,^31^,^32^,^33^,^34^,^35^,^36^,^37^,^38^,^39^,^40^,^41^ are low. The literature quality score is shown in Table 2.

### CK-MB after PCI

**Figure 2:**
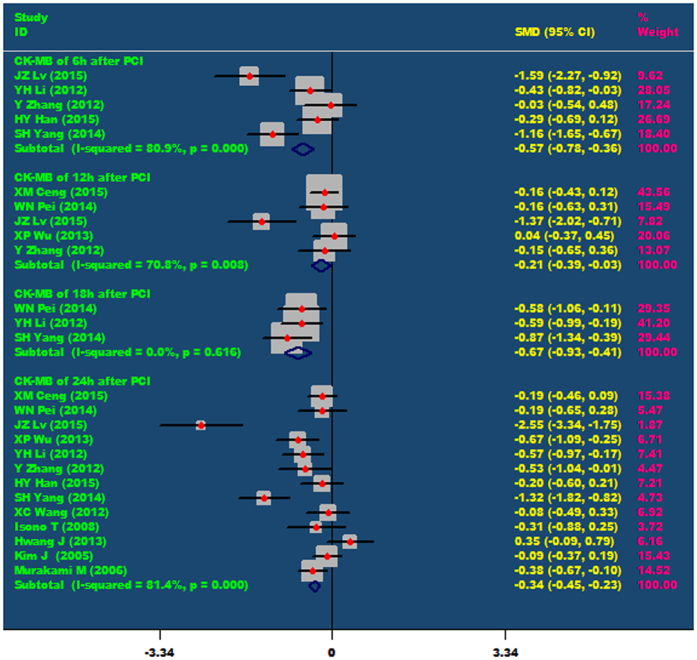
The comparisons of CK-MB at 6 h, 12 h, 18 h and 24 h after PCI between the nicorandil group and the control group.

Nicorandil can significantly reduce the level of CK-MB in patients after PCI, including at 6hours (SMD = −0.57, 95% CI −0.78 ~ −0.36, P < 0.001), 12 hours (SMD = −0.21, 95% CI −0.39 ~ −0.03, P = 0.024), 18 hours (SMD = −0.67, 95% CI −0.93 ~ −0.41, P < 0.001) and 24 hours (SMD = −0.34, 95% CI −0.45 ~ −0.23, P < 0.001) after PCI. As showed in Fig. 2.

### Subgroup analysis

**Figure 3:**
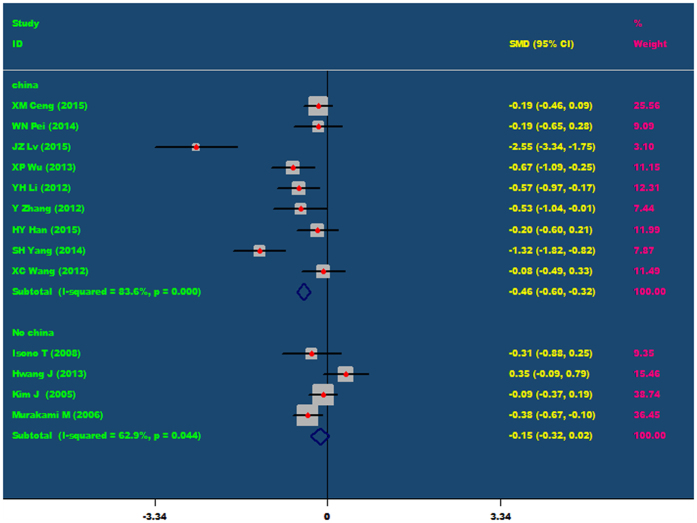
The comparisons of CK-MB at 24 h after PCI between the nicorandil group and the control group (Subgroup analysis: Chinese’s population and non Chinese’s population).

Because the population included in our meta-analysis is mainly coming from China, Japan and Korea, and most of the research objects belong to Chinese population, thus, the population was divided into the Chinese population and non Chinese population, and then conducted a subgroup analysis. Subgroup analysis showed that for Chinese population, Nicorandil can obviously decrease CK-MB level at 24 hours after PCI (SMD = −0.46, 95% CI −0.60 ~ −0.32, P = 0.001). However, for non Chinese population, nicorandil cannot decrease CK-MB level at 24 hours after PCI (P = 0.440), As showed in Fig. 3.

### TnT after PCI

**Figure 4:**
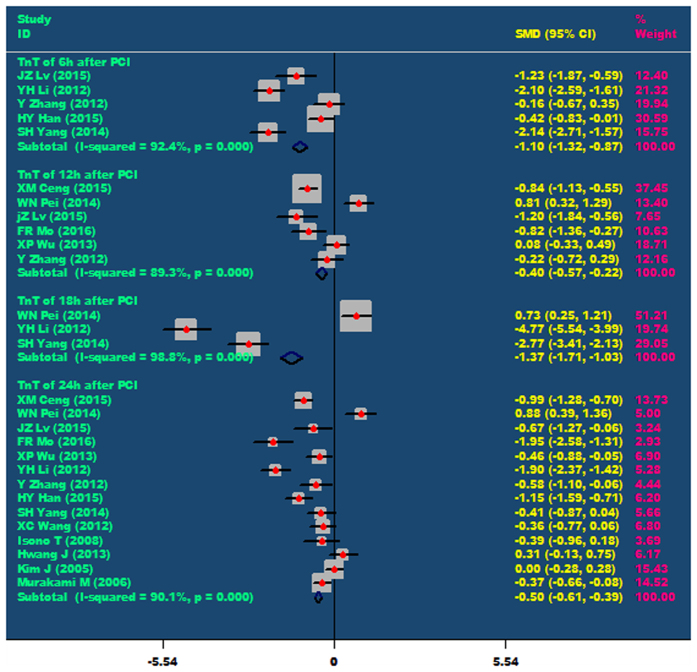
The comparisons of TnT at 6 h, 12 h, 18 h and 24 h after PCI between the nicorandil group and the control group.

Nicorandil can significantly reduce the level of TnT in patients after PCI, including at 6 hours (SMD = −1.10, 95% CI −1.32 ~ −0.87, P < 0.001), 12 hours (SMD = −0.40, 95% CI −0.57 ~ −0.22, P < 0.001), 18 hours (SMD = −1.37, 95% CI −1.71 ~ −1.03, P < 0.001) and 24 hours(SMD = 0.50, 95% CI −0.61 ~ −0.39, P < 0.001). As showed in Fig. 4.

### Subgroup analysis

**Figure 5:**
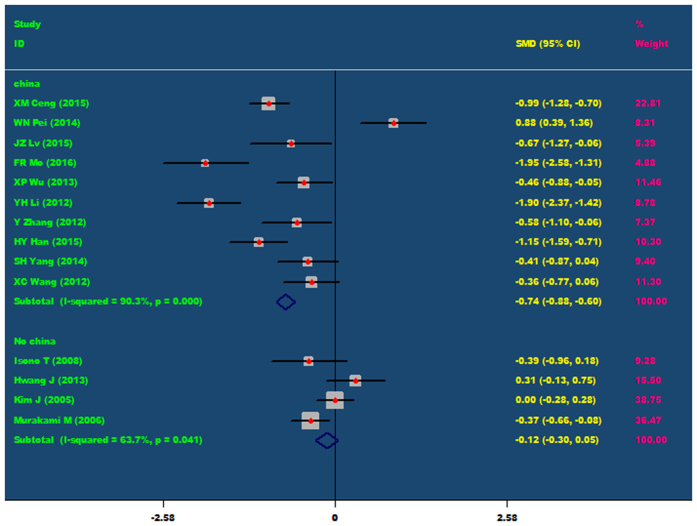
The comparisons of TnT at 24 h after PCI between the nicorandil group and the control group (Subgroup analysis: Chinese’s population and non Chinese’s population).

In addition, all patients were divided into Chinese’s population and non Chinese population, and then conducted a subgroup analysis. Subgroup analysis showed that for Chinese’s people, nicorandil can obviously decrease TnT level at 24 hours after PCI (SMD = −0.74, 95% CI −0.88 ~ −0.60, P = 0.001). However, for non Chinese’s population, nicorandil cannot remarkable decrease TnT level at 24 hours after PCI (P = 0.487), As showed in Fig. 5.

### Incidence of adverse reactions

**Figure 6:**
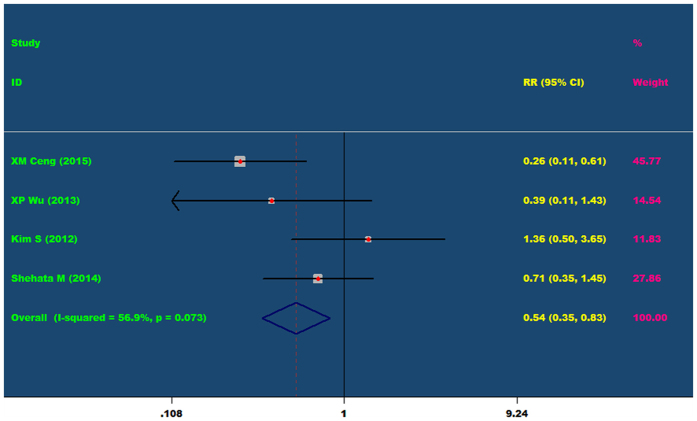
The incidence of adverse reactions in the nicorandil group and the control group (RR = 0.54, 95% CI 0.35~0.83, P = 0.073).

Four articles evaluating the incidence of adverse reactions of nicorandil undergoing elective PCI. Meta-analysis showed that I^2^ = 56.9%, the heterogeneity is large, so random effects model was used to analyze. Meta-analysis (random effect’s model) results showed that nicorandil can reduce the incidence of adverse reactions in patients after elective PCI (RR = 0.54, 95% CI 0.35 ~ 0.83, P = 0.005). As showed in Fig. 6.

### MPV after PCI

**Figure 7:**
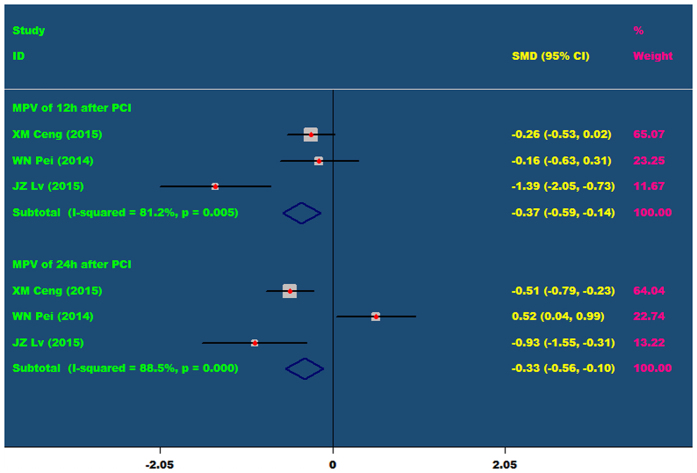
The comparisons of MPV at 12 h and 24 h after PCI between the nicorandil group and the control group.

Nicorandil cannot significantly reduce the level of MVP in patients after PCI, including at 12 hours (P = 0.078) and 24 hours (P = 0.445). As showed in Fig. 7.

### Publication bias

**Figure 8:**
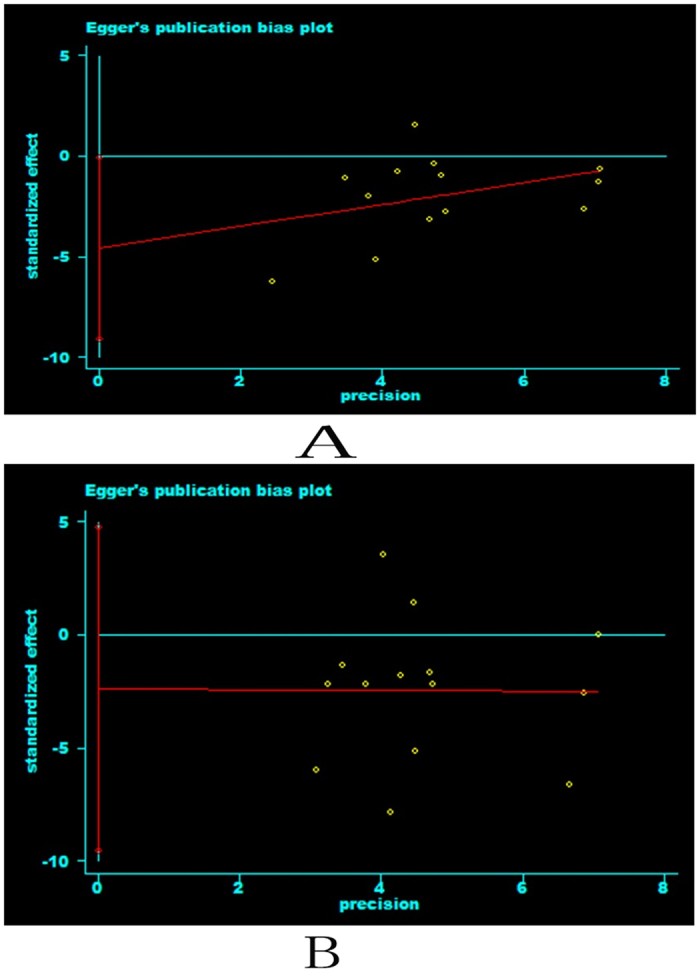
Egger regression analysis was used to evaluate the publication bias. There was no publication bias of CK-MB at 24 hours after PCI (Egger’s test: P = 0.214) (Fig. 8A). There was also no publication biasof TnT at 24 hours after PCI (Egger’s test: P = 0.978) (Fig. 8B).

Egger regression analysis was used to evaluate the publication bias. There was no publication bias of CK-MB at 24 hours after PCI (Egger’s test: P = 0.214) (Fig. 8A). There was also no publication bias of TnT at 24 hours after PCI (Egger’s test: P = 0.978) (Fig. 8B).

## Discussion

Percutaneous coronary intervention (PCI) is one of the most effective methods for the treatment of myocardial infarction (MI) or unstable angina^42^,^43^,^44^. Compared with traditional drug therapy, PCI can more effectively restore the blood supply to the myocardium and improve the prognosis of patients. However, a lot of studies have shown that there are still approximately 3% of patients failing to benefit from the treatment of PCI. On the contrary, the phenomenon of myocardial injury was appeared in a patient after PCI, which seriously affects the patient’s heart function and prognosis. Therefore, how to improve the blood flow perfusion of the ischemic myocardium after PCI and reduce the occurrence of myocardial injury is of particular importance. Research has shown that the K+ -ATP channel opener (such as nicorandil) can significantly reduce arrhythmia, chest pain and slow reflow phenomenon caused by PCI^45^,^46^. However, the conclusion, whether nicorandil has a myocardial protective effect is inconsistent. Thus, we conduct this meta-analysis.

In 2012, Kim S^28^ enrolled 213 consecutive patients with stable or unstable angina, which were expected in non-urgent PCI for de-novo coronary lesions. 54 patients in the nicorandil group and 55 patients in the control group. The purpose of the study is to assess whether nicorandil has a myocardial protective effect or not. Finally, they found that there were no significant differences in the incidence of post-procedural myocardial necrosis among the two groups (10.9% vs 14.8%, respectively, p = 0.9) and there were no significant differences in the incidence of post-procedural MI among two groups (p = 0.6). Meanwhile, in 2013, Hwang J^26^ conducted a clinical experiment includes 41 patients in the nicorandil group (n = 41) and 40 patients in the control group (n = 40). In the nicorandil group before PCI, four mg of intracoronary nicorandil was infused prior, and the results showed that the post-PCI peak CK-MB and troponin I levels were not significantly different between the two groups. However, Isono T^41^ conducted an experiment, including 29 patients undergoing elective PCI. And they found that nicorandil can suppress elevations of cardiac enzymes after elective PCI, suggesting that nicorandil enhances the myocardial protective effect of PCI against angioplasty-related myocardial injury. Kim S^28^ and Shehata M^29^ have also carried out a trial, and they results showed that nicorandil can protect the myocardium, which was different from Hwang J and Kim J. Therefore, it is necessary to make a systematic evaluation of this conclusion. In our meta-analysis, a total of 16 randomized controlled trials (RCTs) were included, and 1616 patients entered our study. Our results showed that nicorandil can reduce the levels of CK-MB and TnT after elective PCI and the incidence of adverse reaction caused by PCI for Chinese’s population, but the clinical benefit of nicorandil is not obvious for non Chinese’s population. Through this meta-analysis, we speculated that nicorandil has a cardioprotective effect for Chinese’s population, but may not be appropriate for non Chinese’s population.

Nicorandil is an ATP sensitive potassium channel (KATP) open agent with the effect of nitrate^47^,^48^. At the present, research on the myocardial protective effect of nicorandil after PCI is intravenous administration. Results have shown that intravenous nicorandil before PCI can significantly reduce the incidence of coronary slow flow after postoperative^49^,^50^. The mechanism may be related with the decrease of infiltration of neutrophils into the ischemic area, which leading to the decrease of neutrophil mediated microcirculation. Our meta analysis results showed that when compared with the control group, the myocardial enzyme levels were significantly decreased in the nicorandil group after PCI, and the myocardial enzyme level of some time point was lower than that of the control group (P < 0.05). Acting as a KATP agent, nicorandil can shorten the action potential duration, inhibit calcium overload. Furthermore, nicorandil can antagonize ADP induced platelet aggregation, improve microcirculation in ischemia area, decrease no reflow phenomenon. In addition, nicorandil can inhibit the formation of active oxygen, which is one of the mechanisms implicated in the protective effect on the myocardium^51^.

The limitations of our meta-analysis include the following aspects: ① The included studies are mainly coming from China, Japan and Korea, lacking of randomized controlled trials from North America and Europe. ② The method of drug delivery is uniform. Some articles adopted the method of oral administration, and some articles adopted the method of intravenous administration, even some articles using the method of coronary administration, which may lead to a certain bias in implementation. In addition, there is no unity for the dosage and using time for Nicorandil. The possibility of implementation bias is further increased. ③Although the articles included in our meta-analysis were RCT, but most of the studies were single-blind RCT, bias will be inevitable appear. ④Short-term and long-term index need to be observed to evaluate the myocardial protective effects of nicorandil in patients after PCI. Because CK-MB, TnT, MPV and adverse reactions all belongs to short-term index, lacking the long-term index (For example, heart failure, myocardial infarction and arrhythmia, etc.) to estimate the effect of nicorandil, the representation is poor.

Our study also suggests that some aspects should be paid attention to in the future when carrying out RCT: ① Due to the difference of nation and race, it needs to conduct RCT of multi-regional and multi-center, in order to evaluate the clinical efficacy of a drug. ② To regulate the use method of drugs, including usage, dosage and use of time, as far as possible to reduce the occurrence of confounding bias. ③ Describe the method of random grouping, single-blind or double-blind and the implementation of the method in detail, so as to reduce confounding bias. ④Long-term indicators should be increased to estimate the clinical effect after PCI, and then increase the reliability of results.

## Conclusion

Our systematic review and meta-analysis indicate that nicorandil can reduce myocardial injury and reduce the incidence of adverse reaction caused by PCI for Chinese’s population, but the clinical benefit of Nicorandil is not obvious for Non Chinese’s population. However, due to the limitations of the quality and quantity of the articles, this conclusion still needs to be confirmed by multi-center, double-blind, randomized controlled trials.

## Methods

### Literature search

According to the statement of the preferred reporting items for Systematic Reviews and Meta-Analyses, two researchers independently searched published randomized controlled trial(RCT) that investigated the clinical effect of nicorandil on prevention of perioperative myocardial injury in patients undergoing elective PCI. The retrieved database includes PubMed, Embase, the Cochrane Library, Web of Science, CBM, CNKI, VIP database and Wang Fang database, the retrieval time was limited from inception to October 7, 2016. Relevant keywords related to nicorandil in combination as MeSH terms and text words (“Nicorandil” or “2-Nicotinamidoethyl Nitrate” or “2 Nicotinamidoethyl Nitrate” or “Nitrate, 2-Nicotinamidoethyl” or “2-Nicotinamidethyl Nitrate” or “2 Nicotinamidethyl Nitrate” or “Nitrate, 2-Nicotinamidethyl” or “SG-75” or “SG 75” or “SG75” or “Ikorel” or “Aventis Pharma Brand of Nicorandil” or “Rhône-Poulenc Rorer Brand of Nicorandil” or “Rhône Poulenc Rorer Brand of Nicorandil” or “Aventis Brand of Nicorandil” or “Nicorandil Aventis Brand” or “Adancor”) were used in combination with words related to percutaneous coronary intervention and myocardial reperfusion injury(“Coronary Intervention, Percutaneous” or “Coronary Interventions, Percutaneous” or “Intervention, Percutaneous Coronary” or “Interventions, Percutaneous Coronary” or “Percutaneous Coronary Interventions” or “Percutaneous Coronary Revascularization” or “Injuries, Myocardial Reperfusion” or “Myocardial Reperfusion Injuries” or “Reperfusion Injuries, Myocardial” or “Myocardial Ischemic Reperfusion Injury” or “Reperfusion Injury, Myocardial” or “Injury, Myocardial Reperfusion”). The retrieval language was limited to Chinese and English. In addition, reference articles of the extracted articles were also retrieved. When multiple reports of the same study were present, we used the most recent publication and supplemented it. All analyses were based on previously published studies, and thus no ethical approval or patient consent was required.

### Study selection

We identified studies that prospectively evaluated the clinical effect of nicorandil on prevention of perioperative myocardial injury in patients undergoing elective PCI. Inclusion criteria:① The study was limited to randomized controlled trials (randomized controlled trials, RCTs), and the purpose of the study was to evaluate the effect of nicorandil on prevention of perioperative myocardial injury in patients undergoing elective PCI; ② At least one of the observation group was applied nicorandil in the experiment; ③The does and usage of nicorandil is not limited; ④The article should provide sufficient data for analysis; ⑤ The study subjects were patients undergoing elective PCI, and coronary angiography was performed; ⑥ The retrieval language is limited to Chinese and English.

Exclusion criteria: ① Retrospective, non-randomized trial; ② Semi randomized controlled trial, in which the grouping method of the participants in the experiment was not strictly random; ③Patients with acute myocardial infarction (AMI) or with AMI within the last 6 months; ④ Articles with incomplete or erroneous data.

### Data extraction

The contents of the retrieved articles were reviewed by two researchers (ZY and QS) in accordance with the prior search methods. Data to be extracted including basic data of subjects (First author, publication year, country, sample number, average age, use method of nicorandil (dose), preoperative method of control group and outcome measures). If there was a lack of necessary data or some content to be clarified in the articles, an effort was made to try to make contact with the study authors, and if the necessary data to analyze was still unavailable, this article was excluded.

### Statistical analyses

We used the Stata software, version 11.0 (Stata Corp, College Station, Tex) to pool and analyze results from the individual studies. Pooled results were reported as relative risks (RRs) and standardized mean difference (SMD), and presented with 95% confidence interval (CI) with two-sided P-values. P < 0.05 indicates that the difference was statistically significant. Heterogeneity of the inclusion study was assessed by I^2^ test, which assessed the appropriateness of pooling the individual study results. When I^2^ < 50%, the heterogeneity of the study was considered small; When I^2^ > 50%, the heterogeneity of the study was considered substantial, and then subgroup analysis and sensitivity analysis were performed to investigate the sources of heterogeneity. If necessary, meta-regression analysis was performed to explore heterogeneity.

## Additional Information

**How to cite this article:** Ye, Z. *et al*. The clinical effect of nicorandil on perioperative myocardial protection in patients undergoing elective PCI: A Systematic Review and Meta-Analysis. *Sci. Rep.* **7**, 45117; doi: 10.1038/srep45117 (2017).

**Publisher's note:** Springer Nature remains neutral with regard to jurisdictional claims in published maps and institutional affiliations.

## Figures and Tables

**Table 1:** Characteristics of studies included in meta-analysis.

Author	year	country	Sample number		Average age (years)		Use method of Nicorandil (dose)	preoperative method of Control group	outcome Measures
			N	C	C	N			
XM Ceng^21^	2015	china	100	100	59.4 ± 10.2	59.4 ± 10.2	Venous pump (12 mg/day)	Conventional therapy	CK-MB,TnT
WN Pe^i^^22^	2014	china	40	32	56.97 ± 9.83	56.97 ± 9.83	Oral (15 mg/day)	Conventional therapy	CK-MB,TnT
JZ Lv^23^	2015	china	25	20	55.41 ± 9.82	55.13 ± 9.62	Oral (15 mg/day)	Conventional therapy	CK-MB,TnT
FR Mo^24^	2016	china	26	31	60.35 ± 12.45	61. 37 ± 10. 98	Oral (15–30 mg/day)	Conventional therapy	TnT
XP Wu^25^	2013	china	48	44	61.3 ± 8.4	61.3 ± 8.4	Oral (15 mg/day)	Conventional therapy	CK-MB,TnT
YH L^i^^26^	2012	china	52	48	66.4 ± 10.2	64.8 ± 10.5	Oral (15 mg/day)	Conventional therapy	CK-MB,TnT
Y Zhang^27^	2012	china	30	30	61.0 ± 5.8	63.0 ± 6.2	Oral (15 mg/day)	Conventional therapy	CK-MB,TnT
HY Han^28^	2015	china	45	49	58.64 ± 8.53	56.93 ± 9.74	Oral (15 mg/day)	Conventional therapy	CK-MB,TnT
SH Yang^29^	2014	china	37	38	65.37 ± 8.24	64.27 ± 8.66	Oral (15 mg/day)	Conventional therapy	CK-MB,TnT
XC Wang^30^	2012	china	42	48	63.47 ± 9.24	63.37 ± 8.06	Oral (15 mg/day)	Conventional therapy	CK-MB,TnT
Shehata M^20^	2014	Egypt	50	50	59.4 ± 7.4	60.2 ± 4.3	Oral (20 mg/day)	Conventional therapy	CK-MB,TnT
Murakami M^19^	2006	Japan	91	101	65.0 ± 9.7	66.1 ± 10.3	intravenous (2 μg/kg/min)	Conventional therapy	CK-MB,TnT
Kim S^18^	2012	Korea	54	55	65.5 ± 7.4	63.2 ± 9.2	Intracoronary (4 mg)	Conventional therapy	CK-MB,TnT
Isono T^31^	2008	Japan	23	26	66.3 ± 7.9	66.5 ± 9.4	intravenous (6 mg/h for 24 h)	Conventional therapy	CK-MB,TnT
Kim J^17^	2005	korea	100	100	60.4 ± 11.7	61.7 ± 8.2	Intravenous (10–15 mg/day)	Conventional therapy	CK-MB,TnT
Hwang J^16^	2013	Japan	41	40	66.2 ± 9	65.3 ± 10	Intracoronary (4 mg)	Conventional therapy	CK-MB,TnT

N: Nicorandil group, C: control group.

**Table 2:** Assessment of Methodological Quality of Included Studies.

Study	Random allocation	Hidden distribution	Blind method	Incomplete Outcome Data	Selective reporting of results	Other bias	quality grade
XM Ceng^21^	Random number method	Low	Single-blind	Low	Low	Low	A
WN Pei^22^	Random number method	Low	Single-blind	Low	Low	Low	A
JZ Lv^23^	Random number method	Low	Single-blind	Low	Low	Low	A
FR Mo^24^	mentioned random	Low	Single-blind	Low	Low	Low	A
XP Wu^25^	mentioned random	Low	Single-blind	Low	Low	Low	A
YH Li^26^	mentioned random	Low	Single-blind	Low	Low	Low	A
Y Zhang^27^	mentioned random	Low	Single-blind	Low	Low	Low	A
HY Han^28^	mentioned random	Low	Single-blind	Low	Low	Low	A
SH Yang^29^	Random number method	Low	Single-blind	Low	Low	Low	A
XC Wang^30^	No clear	Low	No clear	Low	Low	Low	B
Shehata M^20^	block randomization	Low	Single-blind	Low	Low	Low	A
Murakami M^19^	mentioned random	Low	Single-blind	Low	Low	Low	A
Kim S^18^	block randomization	Low	Single-blind	Low	Low	Low	A
Isono T^31^	block randomization	Low	Single-blind	Low	Low	Low	A
Kim J^17^	mentioned random	Low	Single-blind	Low	Low	Low	A
Hwang J^16^	mentioned random	Low	Single-blind	Low	Low	Low	A
